# Hydrochlorate of Phenocoll

**Published:** 1891-08-01

**Authors:** 


### THERAPEUTICS.

### HYDROCHLORATE OF PHENOCOLL.

This substance is chemically the hydrochloric salt of amidoacetoparaphenitidine with the composition—

$$C_6H_4 \left\langle \begin{array}{l} OC_2H_5 \\ NH-OC.\ CH_2-NH_2 \end{array} \right. \text{ or briefly} \left\langle \ C_{10}H_{14}O_2N_2 \right.$$

It is a white powder composed of colourless microscopical crystals, having a bitter taste and soluble in about sixteen times its weight of warm water, forming a neutral solution. Treated with boiling water, this salt crystalises in cubes analogous to those formed by antipyrin. It dissolves in alcohol less freely. It occurs through dehydration of glycocoll and the new compound is, therefore, a phenacetin.

One can separate the pure phenocoll from the hydrochloric salt in the form of crystals combined with one equivalent of water. The melting point of the hydrated base is 95° C. As its solubility in cold water is considerably less than in warm, in fact, is almost nil, the hydrochlorate is utilised. The latter is soluble in alcohol, ether, benzol, and chloroform. By prolonged boiling with weak alkalies it is split up into phenetedin and glycocoll, and the same decomposition results from the action of acids. Professor Robert has stated that this substance is practically non-toxic to animals, and has no injurious effect on the blood, in which it differs from the majority of antipyretics.

Von Mœring has also found that in febrile conditions, in typhus and pneumonia, fifteen grain doses reduce the temperature two degrees without producing collapse or cyanosis, and the antipyretic effect of this dose is about equivalent to twenty to thirty grains of antipyrin, or to about fifteen grains of phenacetin.

Notes of several cases in the clinic of Professor Gerhardt are published by Dr. Hertel, in which this drug was prescribed dissolved in water as an antipyretic and antirheumatic, the doses ranging from seven and a-half to fifteen grains. The results seemed to show that single doses of seven and a-half grains reduce the temperature within about an hour—on an average half a degree Cent. Single doses of fifteen grains reduced the temperature about one and a-half to two degrees in about two hours. With seventy grains in divided doses administered in twenty-four hours the highest febrile temperatures may generally be reduced to normal. No sweating, chills, or collapse were noted. In a few cases of acute articular rheumatism, although antipyrin, salicylate of sodium, and phenacetin had failed in relieving, seventy-five grains of phenocoll, in divided doses, given in the course of twenty-four hours, are said to have relieved the pain in the afflicted joints, but without reducing the temperature.

Phenocoll is reported to have no injurious effect on the kidneys, although the larger doses give a brown colour to the urine. As regards its analgisic effect we have no personal experience, but a medical friend has informed us that a single dose of five grains relieved the lightning pains of a case of locomotor-ataxia, and was much more effectual than larger doses of antipyrin had been.*

* *Bull. Gen. de Thérap.* May 23, 1891; Hertel *Deutsche Medic. Woch.* Apr. 9, 1891.

